# Plasmonic biosensors fabricated by galvanic displacement reactions for monitoring biomolecular interactions in real time

**DOI:** 10.1007/s00216-020-02414-0

**Published:** 2020-01-31

**Authors:** Claudia Pacholski, Sophia Rosencrantz, Ruben R. Rosencrantz, Ruth Fabiola Balderas-Valadez

**Affiliations:** 1 Institute of Chemistry, University of Potsdam, Karl-Liebknecht-Straße 24-25, 14476 Potsdam, Germany; 2 Fraunhofer Institute for Applied Polymer Research IAP, Biofunctionalized Materials and (Glyco)Biotechnology, Geiselbergstraße 69, 14476 Potsdam, Germany

**Keywords:** Optical sensor, Gold nanostructure, Localized surface plasmon resonance, Surface functionalization, Biomolecular interactions, Lectin

## Abstract

Optical sensors are prepared by reduction of gold ions using freshly etched hydride-terminated porous silicon, and their ability to specifically detect binding between protein A/rabbit IgG and asialofetuin/*Erythrina cristagalli* lectin is studied. The fabrication process is simple, fast, and reproducible, and does not require complicated lab equipment. The resulting nanostructured gold layer on silicon shows an optical response in the visible range based on the excitation of localized surface plasmon resonance. Variations in the refractive index of the surrounding medium result in a color change of the sensor which can be observed by the naked eye. By monitoring the spectral position of the localized surface plasmon resonance using reflectance spectroscopy, a bulk sensitivity of 296 nm ± 3 nm/RIU is determined. Furthermore, selectivity to target analytes is conferred to the sensor through functionalization of its surface with appropriate capture probes. For this purpose, biomolecules are deposited either by physical adsorption or by covalent coupling. Both strategies are successfully tested, i.e., the optical response of the sensor is dependent on the concentration of respective target analyte in the solution facilitating the determination of equilibrium dissociation constants for protein A/rabbit IgG as well as asialofetuin/*Erythrina cristagalli* lectin which are in accordance with reported values in literature. These results demonstrate the potential of the developed optical sensor for cost-efficient biosensor applications.

**Electronic supplementary material:**

The online version of this article (10.1007/s00216-020-02414-0) contains supplementary material, which is available to authorized users.

**Figure:**
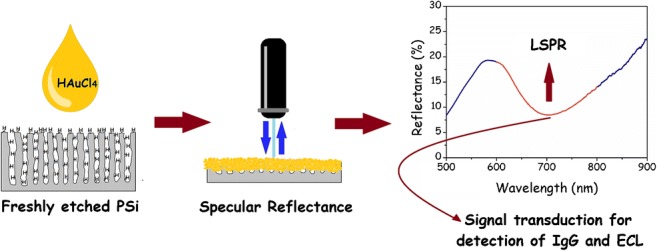
Graphical abstract

## Introduction

The optical phenomenon of surface plasmon resonance (SPR) has been exploited for sensor applications for decades and is based on a collective oscillation of the electron gas in certain materials including gold and silver [1]. Localized surface plasmon resonance (LSPR) in gold nanoparticles, which can be directly excited by light, was found to be highly sensitive to refractive index changes in close proximity to the gold surface (up to ~ 10 nm). It provokes a selective absorption and scattering of photons and the spectral position as well as the intensity of the optical response depend in this case not only on the refractive index of the surrounding medium but also on the size, shape, and material of the metallic nanoparticles facilitating the realization of tailor-made optical sensors [2]. A large variety of fabrication strategies for LSPR sensors have been developed, which can be divided in the employment of either top-down or bottom-up methods as well as a combination of both. Here, top-down methods rely on the fabrication of masks by rather sophisticated methods, for example, e-beam lithography, which facilitate the formation of highly defined nanostructures upon subsequent deposition of gold using physical vapor deposition or other appropriate techniques for creating thin metallic films [3]. Bottom-up strategies are mainly based on wet-chemically synthesized building blocks which are often arranged on appropriate substrates [4] or in hydrogel using self-assembly [5]. However, plasmonic nanoparticles have also been investigated as optical sensors directly in solution by e.g. exploiting controlled aggregation based on chemical interactions [6]. The synthesis of metallic nanoparticles is well-established today and encompasses chemical [7], photochemical [8], and biological routes [9]. The research on LSPR sensors is a very active area and nowadays encompass the optimal arrangement of gold nanostructures on sensor surfaces for providing optimal analyte transport [10, 11], the combination plasmonic structures with other materials (e.g., fluorescent quantum dots for surface-enhanced fluorescence) [12], miniaturization [13], and surface-enhanced Raman spectroscopy leading to the detection of single molecules [14]. However, simple and cost- and time-efficient fabrication strategies for highly sensitive LSPR sensors utilizing shifts in the spectral position of the LSPR for signal transduction were most often fabricated by deposition of wet-chemically prepared gold nanoparticles on substrate surfaces. The benefits of directly using the substrate for preparing plasmonic nanostructures have barely been investigated. One easy and fast approach to do so is based on the growth of metallic nanostructures on semiconductor surfaces via galvanic displacement. The phenomenon was described e.g. by Alia et al. in 2014 [15]. Galvanic displacement occurs when a “template” (metal or semiconductor) comes into contact with a more noble cation (where nobility is related to the standard redox potential). In this case, it is thermodynamically favorable for the more noble cation to “steal” electrons from the less noble template. The galvanic displacement may occur spontaneously or may be promoted by a third agent. For example, in order to promote the growth of gold nanoparticles or thin layers of gold on silicon oxide surfaces, potassium tetrachloroaurate (III) (KAuCl_4_) is offered in solution which also contains a certain percentage of hydrofluoric acid (HF). The parameters of the reaction, the influence of the template orientation, and the proposed reaction mechanism have been nicely reviewed by Lahiri and Kobayashi in 2016 [16]. Similar surfaces may be fabricated by spontaneous galvanic displacement of gold cations on hydrogenated silicon. In this case, the presence of HF was not required. It was proposed that such spontaneous reaction is related to the distribution of charges in the [≡Si-H] group [17]. Nevertheless, even if for none of the two cases the mechanism is completely understood, this technique is useful, reproducible, and for a long time used in industry [18, 19] for the fabrication of ohmic junctions and Schottky barriers. However, the realization of LSPR sensors by galvanic displacement reactions has barely been reported until now, even though substrates for surface-enhanced Raman spectroscopy (SERS) [20–22] or surface-enhanced infrared spectroscopy (SEIRAS) [23, 24] were often prepared in this way.

LSPR sensors are tunable platforms as they detect refractive index changes in close proximity to the sensor surface and are most often used without special labels. Hence, any analyte which has an affinity to the metallic sensor surface will provoke an optical response. To obtain a high selectivity for detecting certain biomolecules and to investigate their binding kinetics, appropriate capture probes for the intended target analyte have to be presented on the sensor surface. For this purpose, different functionalization protocols have been reported ranging from physical adsorption and covalent coupling to complex formation [25]. Between all the possibilities, the functionalization with proteins is, probably, the one that is most often chosen in biosensing experiments due to their extraordinary recognition capacity. For example, glucose oxidase is an enzyme that recognizes and reacts with glucose and commercial sensors are based on this reaction to measure glucose concentrations in blood [26]. One known and useful recognition element for testing the ability of sensors to monitor biomolecular interactions in real time is Protein A (PrA), a protein found in the cell wall of the bacterium *Staphylococcus aureus*, which plays an important role in the virulence of such bacteria by binding to antibodies [27]. The PrA may be bound to metallic surfaces in order to coffer them a high selectivity for antibodies, especially certain immunoglobulin G (IgG). It has been shown that highly sensitive LSPR biosensors can be obtained by well-ordered nanostructures functionalized with PrA [28, 29]. Another application field is the recognition of glycan structures. Glycans are found on every living higher cell and enable communication between a cell and its surroundings [30]. They appear as glycolipids, as proteoglycans, or as glycoproteins. Glycosylation is a complex post-translational modification due to many different enzymes involved in this process. The structural complexity of oligosaccharides allows information-coding. Their counterparts are specific glycan recognition proteins, so-called lectins [31]. In this context, the asialofetuin/*Erythrina cristagalli* lectin interaction can be used as model system. Methods like SPR, ITC (isothermal titration calorimetry), MST (microscale thermophoresis), or ELLA (enzyme-linked lectin assay) can be explored for measuring carbohydrate binding events, on the one hand to understand biological processes and on the other hand for glycan analysis. LSPR sensors using glycopolymers, gold nanoparticles, or self-assembled monolayers are already described for lectin binding studies [32–36]. However, for providing LSPR sensors by mass production, both a well thought through functionalization strategy of the sensor surface with the desired capture probes and a time- and cost-efficient fabrication method are required.

In this work, a simple and fast fabrication strategy for LSPR sensors using galvanic displacement reactions in combination with cost-efficient surface functionalization methods is presented, and the potential of the resulting nanostructured gold layers on silicon substrates for monitoring biomolecular interactions is demonstrated.

## Materials and methods

### Materials

Silicon wafers (p-type, 0.001–0.002 Ω cm, <100>) were obtained from Siegert Wafer GmbH (Germany). Hydrofluoric acid (48%), 2-propanol, and toluene were purchased from Merck. Ethanol (99.8%), glutardialdehyde, and hydroxyethyl piperazineethanesulfonic acid (HEPES) were supplied by Carl Roth GmbH + Co. KG (Germany). Galvanic displacement reactions were carried out in ethanol (96%) supplied by VWR International GmbH (Germany) and with HAuCl_4_ · 3 H_2_O (99.99%) obtained from Alfa Aesar (Thermo Fisher (Kandel) GmbH, Germany). Cysteamine, Protein A, rabbit IgG, PBS buffer, bovine serum albumin, asialofetuin, and acetic acid were supplied by Sigma. *Erythrina cristagalli* lectin was purchased from Vector Laboratories via BIOZOL Diagnostica Vertrieb GmbH (Eching, Germany). Hydrochloric acid (HCl) was supplied by Th. Geyer.

### Fabrication of sensors based on gold nanostructures

First, a sacrificial layer of porous silicon was prepared by electrochemically etching of p-type silicon wafer pieces (0.001–0.002 Ω cm, orientation <100>, Siegert Wafer). Etching was carried out using an electrolyte containing ethanol and hydrofluoric acid at 48% in a ratio 1:1 (v:v). A current density of 133 mA cm^−1^ was applied for 67 s using a Kepco Power Supply. Freshly etched porous silicon samples were immediately immersed in a 2 mM solution of HAuCl_4_ · 3H_2_O dissolved in a 1:2 (w:w) mixture of ethanol and MilliQ water. This gold nanostructures growth reaction was carried out at a controlled temperature of 31 °C. After a reaction time of 7 min, the samples were removed from the gold salt solution, washed extensively with ethanol, and dried in a stream of N_2_. Subsequently, the samples were incubated in basic solution (15 mM NaOH dissolved in a 1:1 (w:w) mixture of ethanol:water) overnight. The basic solution was removed in the morning, and the samples let dry in air. Details of the optimization process for preparing nanostructured gold layers using galvanic displacement reactions can be found in the Electronic Supplementary Material (ESM) of this article.

### Scanning electron microscopy

Scanning electron micrographs were obtained with a Zeiss Ultra 55 “Gemini” scanning electron microscope (Carl Zeiss, Inc., Oberkochen, Germany), which was operated at an accelerating voltage of 10.0 keV. Backscattered electrons were detected for obtaining the presented micrographs.

### Optical characterization

An Ocean Optics, Inc. (USA) charged-coupled device (CCD) spectrometer (model Flame) was utilized for collecting reflectance spectra. For this purpose, a bifurcated optical fiber was equipped with a microscope objective lens and connected to both the spectrometer and a tungsten light source. Through the microscope objective lens a spot with a size of ~ 1–2 mm^2^ was illuminated with light. Reflectivity spectra were recorded from 400–1000 nm with a spectral acquisition time of 5.7 ms and a total integration time of ~ 10 s resulting from averaging five spectral scans. Reflectivity spectra were collected at normal incidence. In order to obtain reflectance spectra, the reflectivity spectrum of the respective sample was divided by a reflectivity reference spectrum which was collected previously from an aluminum mirror.

### Determination of the sensor sensitivity

The bulk sensitivity in nm/RIU (refractive index unit) of the nanostructured gold layer (nAuL) was determined by measuring the shift in the wavelength of the LSPR (minimum of the plasmonic feature in the reflectance spectrum, referred from here as “*λ*_P_”) in response to the refractive index (*n*) of the medium surrounding the sensor. For this purpose, the reflectance spectra of the nAuL were recorded in air (*n* = 1) and after being immersed in three different organic solvents with different refractive index, namely, ethanol (*n* = 1.361), 2-propanol (*n* = 1.375), and toluene (*n* = 1.497). The difference between the position of the LSPR in liquid and air (*λ*_P, *n* ≠ 1_ − *λ*_P, *n* = 1_) was calculated and plotted versus the *n* of the organic solvents. The slope of the linear relationship corresponds to the sensitivity of the sensor.

### Covalent binding of protein A to nanostructured gold

**Fig. 1:**
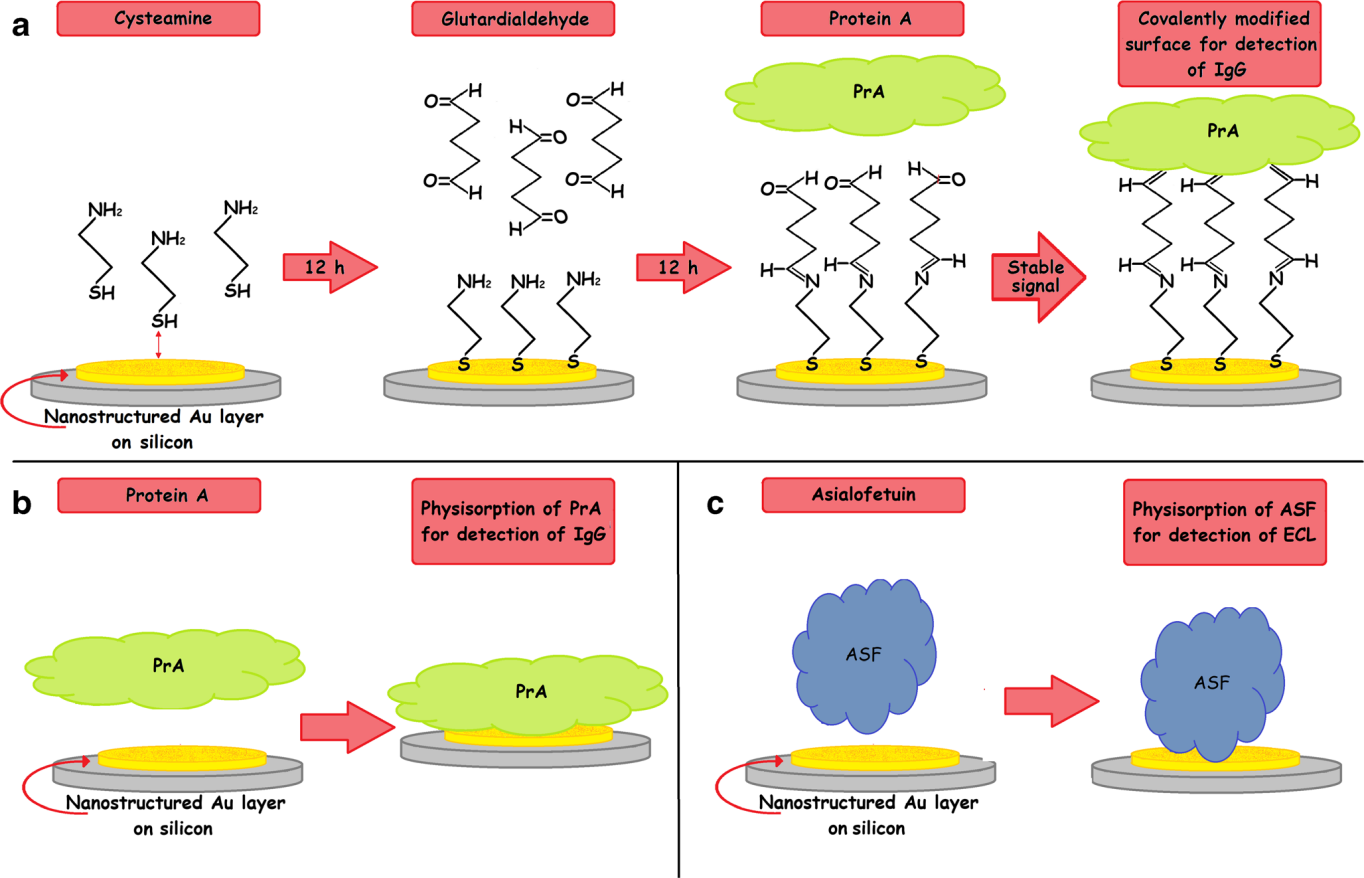
Scheme of the investigated surface functionalization strategies: **a** metallic surface covalently modified with PrA, **b** physisorption of PrA on the nAuL, and **c** physisorption of ASF on the nAuL

Protein A (PrA) was covalently bound to the sensor surface inspired by the method of Boujday et al. [37] and represented in Fig. 1a. For this purpose, the nAuL was incubated in a 10 mM aqueous solution of cysteamine for 12 h, then washed with MilliQ water and dried in a stream of N_2_. The resulting amine-terminated sensor surface was submerged in a 0.1 M ethanolic solution of glutardialdehyde for 12 h, subsequently washed with ethanol, and dried with N_2_. In order to bind PrA, a solution of 0.1 mg ml^−1^ of PrA in PBS buffer was flown over the sensor surface. Details can be found in the following “Real-time monitoring of biomolecular interactions” section.

### Physical adsorption of Protein A to the nanostructured gold layer

PrA was adsorbed to the nAuL without any previous functionalization as is shown in Fig. 1b. For this purpose, a solution of 0.1 mg ml^−1^ of PrA in PBS buffer was flown over the sensor surface. Experimental details of the attachment to the gold surface can be found in the following “Real-time monitoring of biomolecular interactions” section.

### Real-time monitoring of biomolecular interactions

The interaction of biomolecules with the sensor surface was monitored using specular reflectance spectroscopy and by following the shift in *λ*_P_, which is caused by the adsorption or binding of the biomolecule to the sensor surface. For this purpose, the nAuL was fixed inside a custom-made flow cell (plexiglass). Light was guided to and from sensor surface via a bifurcated optical fiber through the plexiglass cover and reflectance spectra were collected in regular intervals. Different solutions were flown over the sensor surface using a peristaltic pump (Perimax 12, SPETEC) and a flow rate of 0.57 ml min^−1^.

For functionalizing the sensor surface with PrA (with and without previous chemical modification), different solutions were successively introduced into the flow cell. First, PBS buffer (pH = 7.4) was flown through the cell for 10 min in order to establish a base line. Afterwards, 5 ml of a PBS buffer solution containing 0.1 mg/ml of PrA (42 kDa) were recirculated through the flow cell. Attachment of the PrA to the nAuL provoked a shift in *λ*_P_. The recirculation of the PrA was continued until the *λ*_P_ did not change anymore. Pure PBS buffer was introduced into the cell for 10 min in order to replace the PrA solution. Then, a 0.1 M aqueous solution of acetic acid was passed over the nAuL to remove PrA from the sensor surface which was not strongly attached to the sensor surface. Finally, the sensor was exposed to PBS buffer again and was ready for biomolecular interaction studies.

The interactions of the PrA-modified sensors with immunoglobulins (IgGs) and bovine serum albumin (BSA) were studied directly after their functionalization. For this purpose, a solution of 0.1 mg/ml of immunoglobulin G (IgG, 150 kDa) from rabbit in PBS was flown through the flow cell, also provoking a shift in the *λ*_P_ due to the association of rabbit IgG to PrA. After obtaining a constant value of *λ*_P_, PBS buffer was passed over the sensor surface in order to monitor the dissociation of rabbit IgG from PrA. To recover the sensor surface, a 0.1 M aqueous solution of acetic acid was introduced into the flow cell, followed finally by an exposure to PBS buffer. This cycle was repeated 4 times using different concentrations of IgG. As negative control, a solution of 1 mg/ml of BSA in PBS buffer was flown over the sensor surface.

### Biomolecular interaction of the system ASF/ECL

In order to demonstrate the capability of the developed optical sensor to also monitor other biomolecular interactions, a second capture probe/target molecule combination, namely, asialofetuin (ASF, 48 kDa) and Erythrina cristagalli lectin (ECL, 54 kDa), was investigated (Fig. 1c). For this purpose, freshly prepared nAuL sensors without functionalization were fixed inside the custom-made flow cell and reflectance spectra were recorded in real time again. The sensor was first exposed to lectin buffer (10 mM HEPES, 150 mM NaCl, 0.1 mM CaCl2, pH 7.5) for 10 min. Afterwards, a solution of 0.25 mg/ml of the glycoprotein ASF in lectin buffer was recirculated through the flow cell until a stable value of *λ*_P_ was observed. Then, the ASF solution was replaced with a solution of 1 mg/ml of BSA in lectin buffer. To remove loosely attached proteins from the sensor surface, a 0.1 M aqueous solution of HCl was passed over the sensor. The interaction of ECL with ASF was then monitored by successively introducing the following solutions into the flow cell: lectin buffer, ECL in different concentrations (dissolved in lectin buffer), lectin buffer, 0.1 M aqueous HCl, and lectin buffer.

## Results and discussion

### Fabrication and characterization of plasmonic sensors

**Fig. 2:**
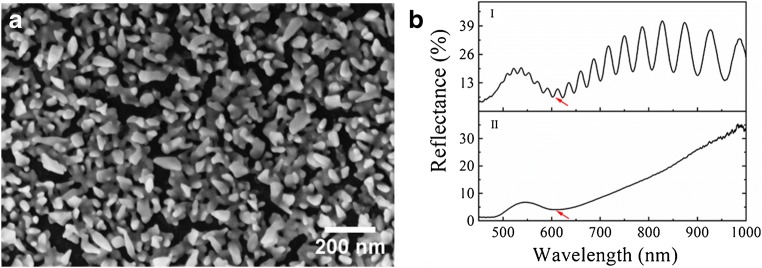
Characterization of fabricated plasmonic sensors: **a** SEM of the nAuL on top of the porous silicon layer, **b** reflectance spectra of a nAuL with (I) and without (II) porous silicon underneath

In Fig. 2a, a representative scanning electron micrograph (SEM) of a fabricated nAuL is displayed. The nAuL was obtained by immersing freshly etched porous silicon in a solution containing Au^3+^ ions. Here, hydrides covering the surface of freshly etched porous silicon reduce Au^3+^ ions leading to the formation of a nanostructured gold layer on top of the porous silicon. The formed nAuL is composed of polydisperse nanoparticles with different shapes, which are mainly non-spherical. Moreover, longer gold nanorods are randomly dispersed over the whole surface. The growth of similar gold nanostructures on silicon by galvanic displacement reactions was already reported [16, 38, 39]. The gold nanostructures were exclusively located on the top surface of the porous silicon layer and no gold could be observed in the pores by inspecting cross-sectional SEMs. The appearance of this nAuL did not change after the removal of the porous silicon underneath using a basic solution.

The formation of nanostructured gold layers on freshly etched porous silicon was monitored by collecting reflectance spectra at normal incidence. A reflectance spectrum of a freshly etched porous silicon layer is characterized by an interference pattern resulting from the superposition of light rays reflected at the interfaces of the porous silicon layer. After immersion of the freshly etched porous silicon in a solution containing Au^3+^ ions, gold nanoparticles start to grow at the porous silicon surface leading to the appearance of a valley in the interference pattern. In Fig. 2b (I), a representative reflectance spectrum of a porous silicon layer with gold nanostructures on top after an immersion time of 7 min is displayed. The valley located at ~ 600 nm and indicated with the red arrow is caused by the excitation of localized surface plasmon resonance in the deposited gold nanoparticles [21]. If the porous silicon underneath the gold nanoparticle layer is removed by dissolution in basic solution, only this valley was observed in the reflectance spectrum (red arrow in Fig. 2b (II)) in accordance with published spectra for gold nanoparticles on silicon substrates [40].

### Plasmonic sensor characterization and functionalization

**Fig. 3:**
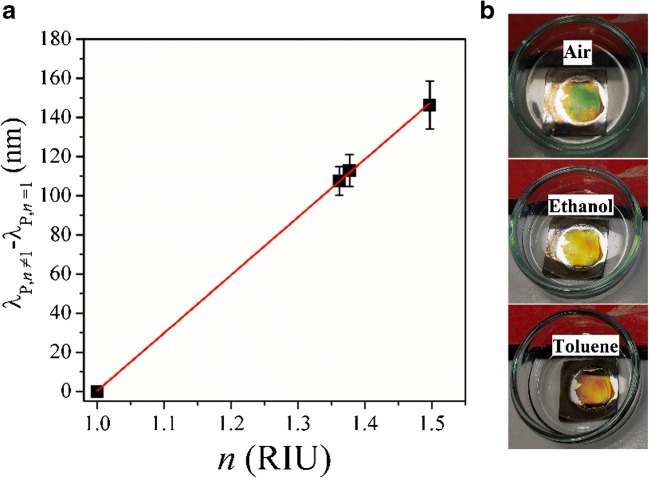
Properties of plasmonic sensors: **a** determination of the bulk sensitivity by plotting the spectral position of the LSPR signal versus the refractive index of the surrounding medium, **b** photographs of the plasmonic sensor in air and organic solvents with different *n*

The position of the characteristic LSPR signal in the reflectance spectrum shifts to smaller or longer wavelengths depending on the refractive index of the surrounding medium. The bulk sensitivity of the plasmonic sensor can be obtained by considering the magnitude of this shift and is quantified in nm/RIU (refractive index unit). In Fig. 3a, the difference between the spectral position of the LSPR signal resulting from the nAuL immersed in liquids with different refractive indices and the spectral position of the LSPR signal of the gold nanoparticle layer in air (“*λ*_P, *n* ≠ 1_ − *λ*_P, *n* = 1_) is plotted versus the refractive index of the surrounding medium. Seven different plasmonic sensors were investigated for this purpose and a bulk sensitivity of 296 nm ± 3 nm/RIU was determined by calculating the slope of the linear relationship. The determined sensitivity is in the upper region for LSPR-based optical sensors [41]. The small standard deviation indicates the high reproducibility of the presented fabrication strategy for plasmonic sensors using galvanic displacement reactions. Also, the shift in the *λ*_P_ due the change in the *n* of the surrounding medium can be observed by the naked eye. Photographs of the plasmonic sensors immersed in different liquids are displayed in Fig. 3b. In air (*n* = 1), the structure shows a vivid green color; after being immersed in ethanol (*n* = 1.361), the color of the surface changed to yellow and in toluene (*n* = 1.497) to a reddish color.

**Fig. 4:**
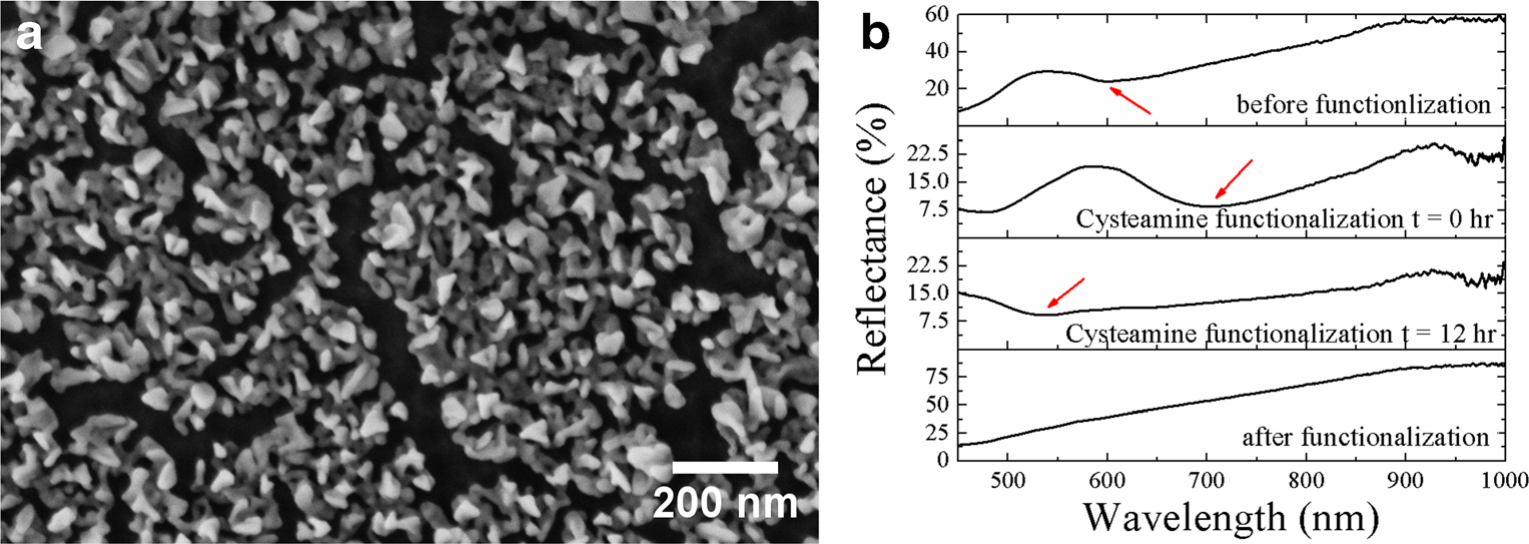
Characterization of the nAuL after cysteamine functionalization. **a** SEM of the gold nanostructures after functionalization with cysteamine, and **b** reflectance spectra of plasmonic sensor before and after its functionalization with cysteamine. Red arrows highlight the position of the LSPR feature

To obtain an amino-terminated sensor surface suitable for covalent binding of biomolecules using glutardialdehyde, the gold nanoparticle layer was immersed in a 10 mM aqueous solution of cysteamine for 12 h. Cysteamine forms a self-assembled monolayer on the nAuL in which the thiol is bound to the gold and the amine group is presented to the surrounding medium [42]. In Fig. 4a, a representative SEM of a nAuL after cysteamine treatment is shown. In comparison to the nAuL before functionalization (Fig. 2a), the gold nanostructures size significantly decreased and the morphology of the nanostructures appears smoother (for better comparison please check the ESM: Fig. S6). Similar observations have been reported for gold nanoparticles immersed in solutions with high cysteamine concentrations [43, 44]. The reduction of the gold nanoparticle size in the fabricated plasmonic sensors leads also to changes in their optical properties—as expected. The spectral position of the LSPR signal in the reflectance spectrum of the plasmonic sensor is shifted to lower wavelengths as shown in Fig. 4b. In air and before functionalization, the plasmon resonance is located approximately at 600 nm. After immersion of the sensor in an aqueous solution of cysteamine, the LSPR shifted to a *λ*_P_ ≈ 700 nm as is expected due the change in the *n* of the surrounding medium. After 12 h incubation in the cysteamine solution, the plasmon resonance can be found to be at a *λ*_P_ ≈ 540 nm (still immersed in aqueous solution). The changes in the spectral position of the LSPR can be caused by a reduction in the size of the gold nanostructures. However, after the functionalization and when the structure is dried, the valley in the reflectance spectra, caused by LSPR, cannot be easily observed anymore. This might be explained by a further shift of the LSPR resonance to shorter wavelengths which have not been investigated. Nevertheless, if the refractive index in the surrounding medium changes from air to e.g. buffer solution, the LSPR can be well detected again. Finally, the amine-terminated sensor surface was submerged in a 0.1 M ethanolic solution of glutardialdehyde for 12 h in order to allow for covalently coupling of biomolecules. In this case, free aldehyde groups react with amine groups present in the biomolecules.

### Real-time monitoring of biomolecular interactions

Two different model systems were investigated, namely, protein A (PrA)/rabbit IgG and asialofetuin (ASF)/ *Erythrina cristagalli* lectin (ECL), for studying the performance of the fabricated plasmonic biosensor regarding real-time monitoring of biomolecular interactions. PrA was first isolated from the cell wall of the bacteria *Staphylococcus aureus* and can bind antibodies [27]. Therefore, it was and still is often utilized for the separation, immobilization and detection of immunoglobulins. ECL is a plant lectin with a high selectivity for binding to carbohydrate moieties of glycoproteins and glycolipids [45]. Lectins are responsible for cellular recognition, adhesion, signal transduction, and metastasis. ASF is a standard glycoprotein presenting terminal galactose residues that can be bound by lectins like ECL [46].

#### Protein A—rabbit IgG interactions

**Fig. 5:**
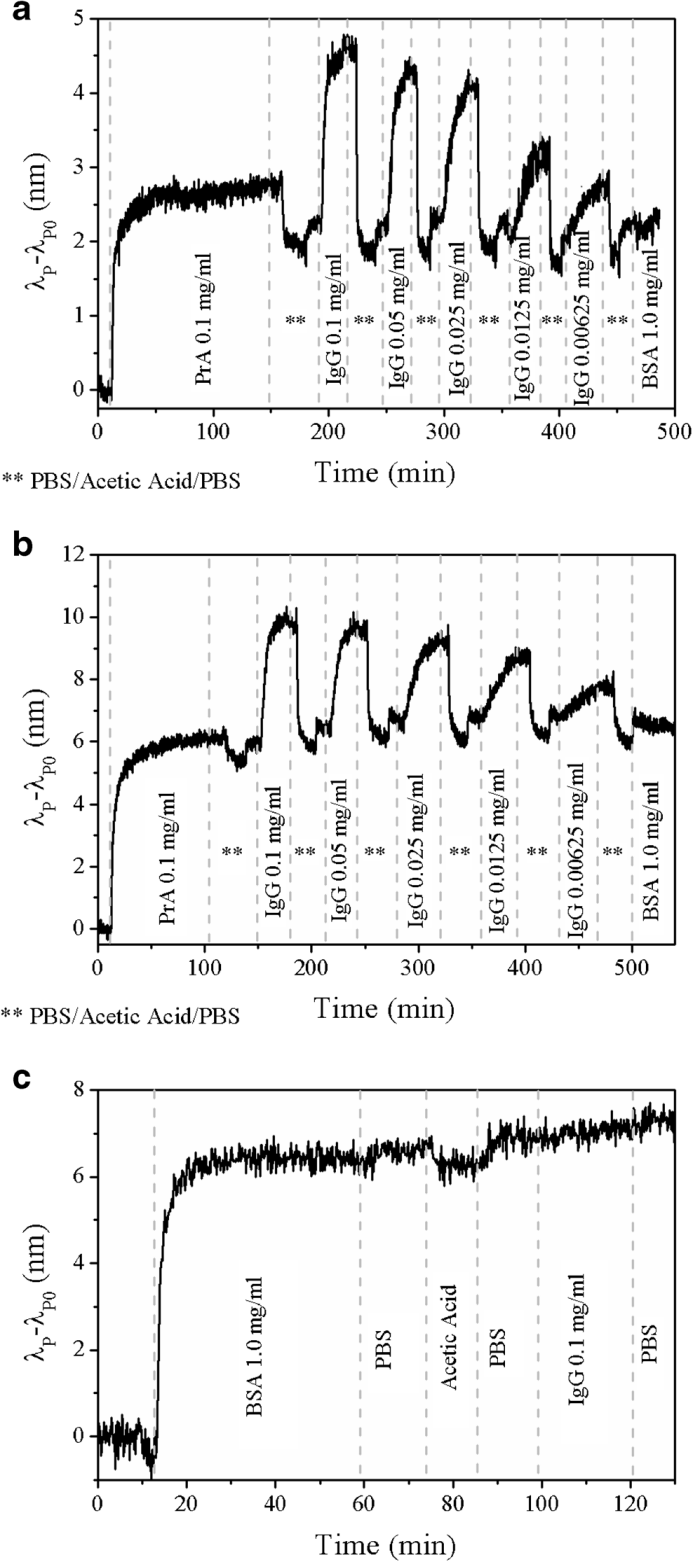
Optical response of plasmonic sensors to deposition of PrA and successive exposure to rabbit IgG solutions: **a** results for plasmonic sensor with covalently bound PrA, **b** results for plasmonic sensor with physically adsorbed PrA, **c** observed spectral shifts of plasmonic sensors with physically adsorbed BSA

First, the biosensor surface had to be equipped with PrA. For this purpose, plasmonic sensors with and without cysteamine/glutardialdehyde functionalization were treated in the same way. Both were fixed in a custom-made flow cell and successively exposed to different solutions, namely, PBS buffer, a solution of 0.1 mg/ml PrA, PBS buffer, 0.1 M aqueous acetic acid solution, PBS buffer. The deposition of PrA was followed by collecting reflectance spectra and following the spectral position of the LSPR signal (Fig. 5).

The optical response of the plasmonic sensor to covalent coupling of PrA to the sensor surface functionalized with cysteamine/glutardialdehyde is shown in Fig. 5a. In the first 10 min, PBS was flown over the sensor surface in order to establish a stable baseline. Afterwards, PrA was introduced into the flow cell leading to a shift in the spectral position of the LSPR signal due to an increase of the refractive index at the sensor surface. The magnitude of the shift was determined in triplicate—from three different plasmonic sensors—to be 2.8 nm ± 0.2 nm demonstrating the high reproducibility of the fabrication process as well as of the functionalization strategy. The PrA solution was recirculated through the flow cell until no significant shift in the LSPR signal position could be detected anymore. Then, the sensor was exposed to an aqueous 0.1 M acetic acid solution in order to remove unbound PrA. The spectral position of the LSPR signal shifted to shorter wavelengths indicating the removal of adsorbed PrA from the sensor surface. Finally, PBS buffer was introduced into the flow cell leading to a small optical response of the sensor which is based on the difference between the refractive index of PBS buffer and of aqueous 0.1 M acetic acid solution. Overall, the covalent binding of PrA to the sensor surface previously functionalized with cysteamine and glutardialdehyde resulted in a change of ~ 2 nm in the spectral position of the LSPR signal (sensor immersed in buffer before and after deposition of PrA). Similar experiments were carried out using plasmonic sensors without functionalization, i.e., PrA was adsorbed directly to the gold surface. The optical response of the sensor to PrA adsorption is shown in Fig. 5b. A shift of 7.0 nm ± 0.7 nm in the spectral position of the LSPR signal was determined from triplicate measurements. We speculate that the difference in the magnitude of the shifts due to the deposition of PrA by physical adsorption or covalent attachment on the plasmonic sensors can be explained by the distance of the biomolecule to the sensor surface and the changes in the gold nanostructure caused by reaction with cysteamine. In the first case, cysteamine/glutardialdehyde increase the distance of PrA to the gold surface. As the electromagnetic field of LSPR has a very short, exponentially decreasing decay length, this additional distance between gold surface and PrA should be one explanation for the smaller shift [47]. Furthermore, the treatment of the gold nanostructure with cysteamine changed its morphology leading to a shift of the LSPR signal to shorter wavelengths and to a somehow smoother gold surface. Both could result in a lower sensitivity of the plasmonic sensor due to the wavelength dispersion of the refractive index and the available surface area.

Directly after deposition of PrA, the plasmonic sensors were exposed to solutions with different concentrations of rabbit IgG in PBS buffer in order to evaluate the capability of the sensors to monitor biomolecular interactions in real time. PrA is known to bind strongly to rabbit IgG [48]. The optical response of the plasmonic sensors functionalized with PrA either by covalent binding or physical adsorption showed an optical response to the presence of rabbit IgG (Fig. 5a, b, respectively). Here, the magnitude of the spectral shifts of the LSPR signal depended on the chosen functionalization strategy and on the concentration of the antibody in PBS buffer: at higher concentrations of rabbit IgG, the shift was larger in comparison to lower concentrations of rabbit IgG. The difference in the observed spectral shifts for plasmonic sensors with covalently bound or physically adsorbed PrA can be explained in a similar way as before described in detail for the deposition of PrA. To test for non-specific binding, a solution of BSA in PBS buffer was also flown over the sensor surface which did not lead to changes in the spectral position of the LSPR signal demonstrating the selectivity of the plasmonic sensors. Also, it is important to highlight that even if the spectral shifts due to the biomolecular interactions at the sensor surface are higher for the plasmonic sensor with physically adsorbed PrA, the stability of its base line, i.e., the ability to return to the same spectral position after the association, dissociation, and removal of rabbit IgG from the sensor surface, is better for the plasmonic sensor with covalently bound PrA. An explanation for this behavior might be based on the prevention of detachment of PrA during drastic changes in pH by the covalent bonds between PrA and sensor surface. In the case of physically adsorbed PrA, desorption might occur during the experiment, opening up space for non-specific binding between the sensor surface and rabbit IgG [25]. It is noteworthy, that in the case of covalent binding using glutardialdehyde it is often highly recommended to block non-reacted aldehyde groups with ethanolamine or glycine in order to prevent non-specific binding of analyte molecules to the sensor surface [49].

A negative control was carried out by physically adsorbing BSA to the plasmonic sensor surface and by successively flow a solution with a high concentration of rabbit IgG in PBS buffer over its surface. In Fig. 5c, the optical response to both the deposition of BSA and the exposure to rabbit IgG are shown. As expected, the adsorption of BSA to the sensor surface resulted in a significant spectral shift of the LSPR signal due to the change in the refractive index caused by BSA. Exposure of the plasmonic sensor to rabbit IgG did not result in an optical response indicating that the spectral shift of the LSPR signal caused by rabbit IgG in Fig. 5a, b is due to its binding to PrA instead of changes in the refractive index in the surrounding medium or non-specific interactions between the sensor surface and rabbit IgG.

**Fig. 6:**
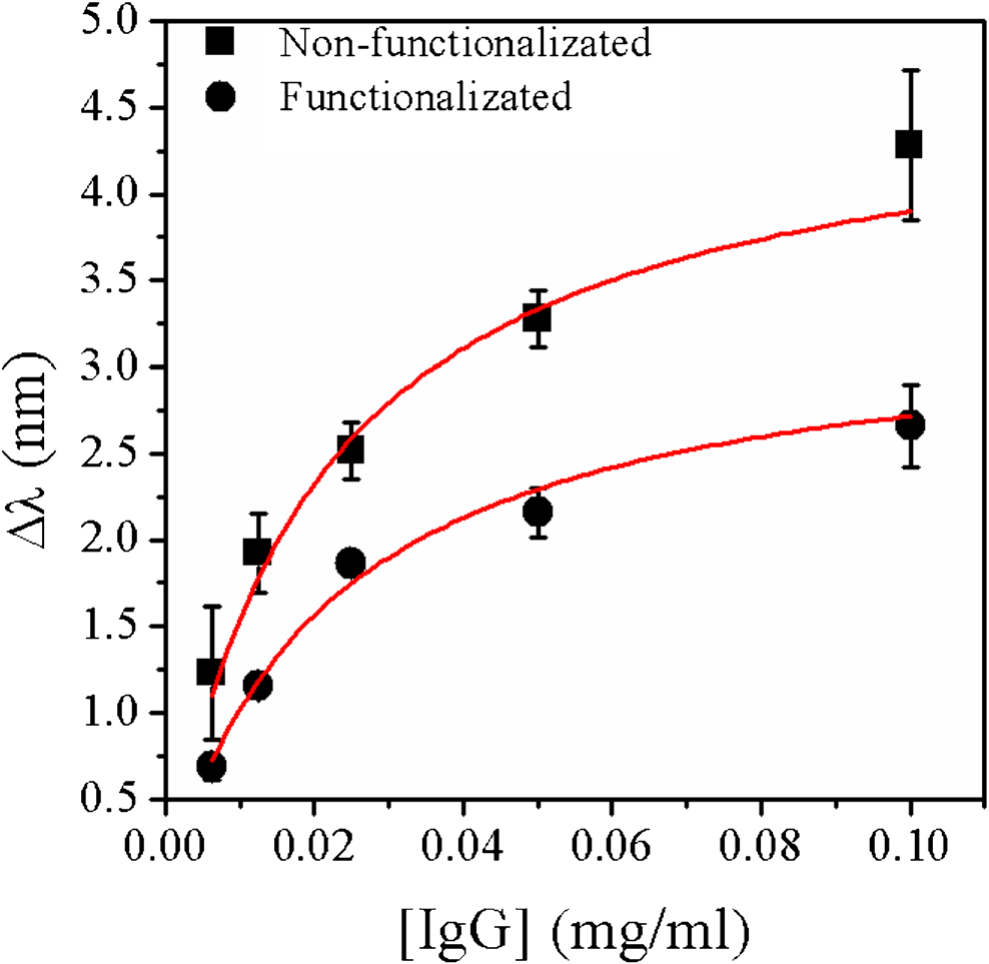
Dependency of the spectral position of the LSPR signal on the offered concentration of rabbit IgG in PBS buffer. Values are shown for three trials using three separately fabricated plasmonic sensors which were functionalized with PrA by physical adsorption or covalent coupling. Solid lines correspond to fitting these values to a Langmuir isotherm for determining equilibrium dissociation constants (*K*_D_ values)

The sensorgrams also provided information on the association and dissociation of rabbit IgG to/from protein A and facilitated the kinetic analysis of the biomolecular interaction in order to extract thermodynamic equilibrium binding constants. For this purpose, the spectral shift of the LSPR signal was determined by:

$$ \Delta  \uplambda ={\uplambda}_{IgG}-{\uplambda}_0 $$

where *λ*_0_ is the spectral position of the LSPR signal after deposition of PrA in PBS buffer and *λ*_IgG_ is the spectral position of the LSPR signal at equilibrium of rabbit IgG binding to the sensor surface at different concentrations. In Fig. 6, ∆*λ* is plotted versus the concentration of rabbit IgG for plasmonic sensors functionalized by physical adsorption or covalent coupling of PrA to the sensor surface. For extracting the equilibrium dissociation constant *K*_*D*_, the average values of three trials are fitted to a Langmuir isotherm [50, 51] resulting in values of *K*_*D*_ = 1.5 × 10^−7^ M ± 2.7 × 10^−8^ M and *K*_*D*_ = 1.4 × 10^−7^ M ± 2.8 × 10^−8^ M for the sensors functionalized by covalent coupling or physical adsorption of PrA, respectively. These *K*_D_ values are in accordance with reported ones for similar studies [52].

#### ASF-ECL interactions

**Fig. 7:**
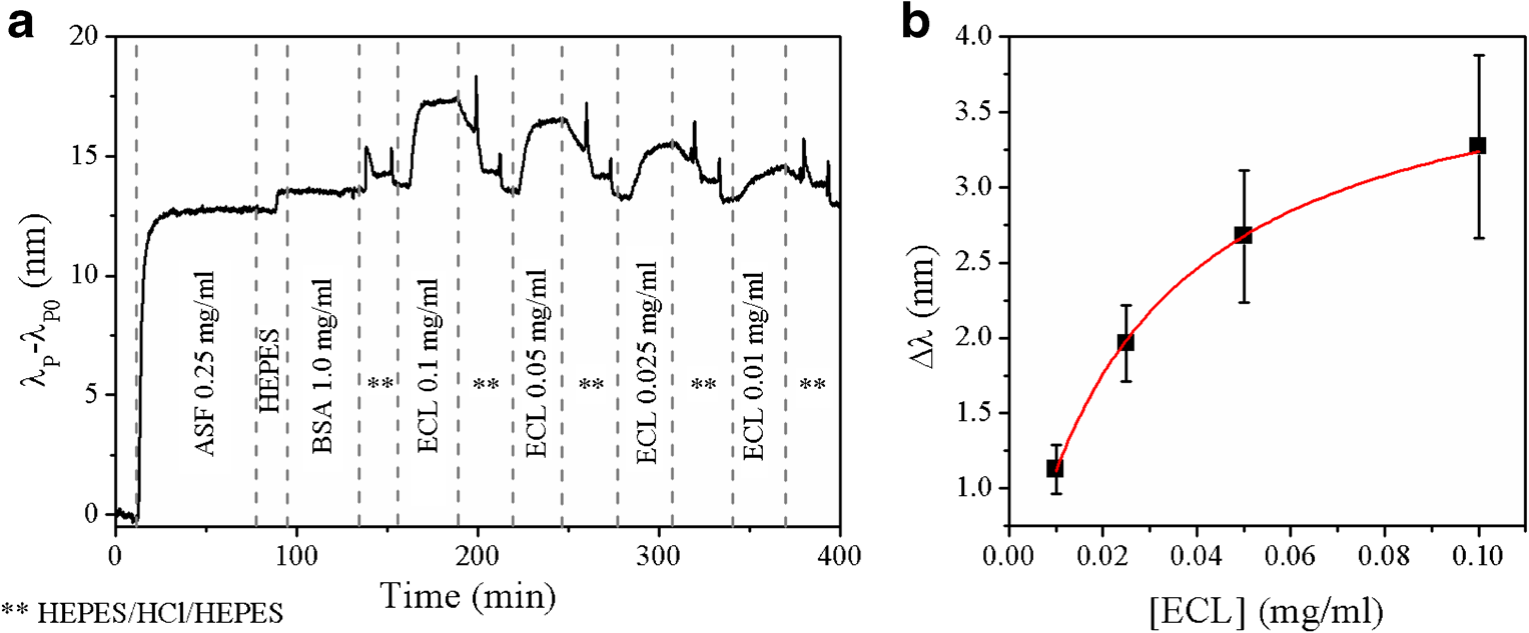
Investigation of ASF-ECL interactions using the fabricated plasmonic sensor: **a** optical response of the sensor to exposure to different solutions. **b** Dependency of the spectral shift of the plasmonic sensor on the concentration of ECL in solution. The solid line represents a fit to a Langmuir isotherm for determining the equilibrium dissociation constant *K*_D_

To demonstrate the generality of the capabilities of the presented plasmonic sensor to monitor biomolecular interactions in real time, another model system, namely, ASF/ECL, was also investigated. Biosensor experiments were performed again by collecting reflectance spectra of the sensor during exposure to various solutions in a flow cell. To avoid size reduction of the nanostructures in the nAuL and a decrease in the response of the sensor [53], no previous chemical functionalization was performed for this system and the attachment of the glycoprotein to the metallic surface was achieved by physical adsorption. The spectral shifts of the LSPR signal indicate the interactions of biomolecules with the sensor surface (Fig. 7a). The spectral shift due to the physical adsorption of ASF to the metallic sensor surface was 11.9 nm ± 0.9 nm. As was expected, BSA did not provoke an optical response of the plasmonic sensor because it does interact with the glycoprotein. The association of ECL to ASF led to spectral shifts of the LSPR signal whose magnitude was related to the concentration of the lectin in the solution. The dependency of the spectral shifts on the concentration of ECL in solution is plotted in Fig. 7b for three trials using three separately fabricated plasmonic sensors. A *K*_D_ value of 4.9 × 10^−7^ M ± 1.7 × 10^−8^ M was determined for ASF/ECL by fitting the data to a Langmuir isotherm, consistent with published values on ASF [54] and on lactose glycopolymers [55].

### Detection limits of the sensors

The smallest amount of analyte that can be accurately detected and quantified by a sensing device is known as the detection limit (DL). The DL should be expressed in units of concentration (M, g L^−1^, etc.) and represents one of the most important performance parameters of a sensor. To evaluate such parameter for the nAuL, the methodology recommended by the IUPAC and explained for Chiavaioli et al. [56] was chosen. This methodology requires a calibration curve of the response of the sensor vs. the concentration of the target analyte at a concentration of 1 to 5 times higher than the suspected DL, an accurate fitting function of such calibration curve, the standard deviation (*σ*) of the blank measurements (measurement without the analyte under investigation), and the mean value of the blank measurement (*Y*_blank_). Then, the concentration of the detection limit is equal to the inverse of the fitting function evaluated in (*Y*_blank_ + 3*σ*). The nanostructured gold layers were evaluated for the detection of IgG using a modified surface of PrA attached either covalently or physically adsorbed to the metallic surface and ECL using a surface modified with ASF. The DL for the three systems were calculated using as calibration curve the data of Figs. 6 and 7b and as fitting function the Langmuir isotherm. The *Y*_blank_ has a normalized value of 0. The magnitudes of the σs are 0.1 nm and 0.2 nm, when nanostructured gold layers with and without cysteamine functionalization were used, respectively. The detection limits were calculated to be 0.4 μg/ml for rabbit IgG detection using covalently coupled protein A on the sensor surface, 0.3 μg/ml for sensors modified with PrA by adsorption, and 0.2 μg/ml for ECL.

Hybrid sensors containing surfaces showing LSPR, which are coupled to optical fibers and report DL in the order of magnitude of nM or fM, have been reported in recent years. However, their optical setup differs considerably from the one used in the presented nAuL sensor. Table 1 lists newer LSPR sensors that can be compared to the nAuL sensor. First of all, it is important to emphasize that such a comparison can be difficult, since the evaluation of the different parameters as sensitivity or the DL can be calculated by different methods. The unit of sensitivity of a plasmonic system is typically given in nm/RIU, but depending on the reference, it can also be given as rad/RIU [59] or nm/nm *** [60]. Another parameter that the DL can influence is the chosen transduction method. For example, Al Rubaye et al. presented in 2017 [57] gold nanoislands fabricated by annealing gold films that exhibit a maxima sensitivity of 77 nm/RIU using absorbance and 207 nm/RIU using spectroscopic ellipsometry. In 2019 [59], the same group reported the exploitation of the same kind of sensor surfaces to achieve a higher sensitivity and a lower DL for the detection of mycotoxin by investigating their performance using a combination of total internal reflection ellipsometry (TIRE), LSPR transducer, and planar waveguides operating as polarization interferometer.

**Table 1:** LSPR sensors

Structure	Analysis methodology	Target	Sensitivity	DL	Reference
Gold nanoislands	Absorbance and TIRE	Mycotoxins as AFT B1	77.28 and 207 nm/RIU*	0.01 ng/ml**	[57, 58]
Gold nanoislands	TIRE/LSPR (absorption)	Mycotoxins as AFT B1	5300 rad/RIU	0.002 ng/ml	[59]
Gold arrays fabricated by interferometric lithography.	Absorbance	Histidine-tagged green fluorescent protein (His-GFP) and bacteriochlorophyll a (BChl a)	145 nm/RIU	–	[60]
Square array of holes coupled with an optical cavity.	Reflectance	Iron oxide nanoparticles (80/100 nm) and Avidin	0.5 nm/nm***	10–3 pM and 0.5 nM	[61]
Square array of holes functionalized with oxygen-deficient cerium oxide nanoparticles	Reflectance	Dopamine in blood	–	1 nM	[62]

*Calculated by absorbance

**Obtained by TIRE

***Analysis made using the shift of the LSPR with respect to the controlled deposition of thin layers of Al_2_O_3_

Since comparing the performance of different sensors is nowadays mainly achieved by evaluating determined DLs, it is crucial to be aware of the different parameters affecting these values. For example, even today, there are discrepancies about the units and the methodology for the calculation of DLs for a wide range of sensing platforms. Some authors use the equation proposed by White and Fan in 2008 [63] that describes the DL as the resolution of the experiment divided by the sensitivity. Other authors claim that such an approximation is not accurate [64] and that the calculation of DLs should be based on calibration curves. Furthermore, such calibration curves should be preferentially measured using analyte concentrations roughly 1 to 5 time higher than the suspected DL. These requirements resulted from the fact that in literature, commonly lower DL were reported which were calculated from experiments carried out with analyte concentrations much higher than the reported magnitude. Also, another important factor, that has a great repercussion on the magnitude of the DL, is the data treatment. Processes like smoothing reduce the noise of the system. Hence, the standard deviation of the measurements is reduced. Moreover, some authors have demonstrated that the DL of a sensor may be enhanced not by the modification of the transduction material but by the modification or change of the data treatment [65]. Finally, the flexibility and capacity of the external instrumentation (e.g., spectrometers) as well as the parameters used in the detection experiment (acquisition time, integration time, light source, etc.) may have an influence on the determined DL. For all these reasons, DLs alone cannot be easily used for comparing the performance of optical sensors.

## Conclusions

A simple and inexpensive optical biosensor capable of monitoring biomolecular interactions was fabricated by the spontaneous galvanic displacement reaction of Au^3+^ cations on freshly etched porous silicon covered with hydrogen groups. The reduction of the metal ions led to the formation of a continuous nanostructured gold layer on the porous film. In the reflectance spectrum of the gold/porous silicon hybrid structure, a broad valley at ~ 604 nm in air in the interference pattern was observed, which was resulting from the excitation of localized surface plasmon resonance in the gold nanostructures. The isolation of the nanostructured gold layer was achieved by dissolution of the porous silicon matrix using basic solution. The remaining nanostructured gold layer showed a high sensitivity to refractive index changes in the surrounding medium. Changes in the localized surface plasmon resonance were exploited for studying biomolecular interactions in real time. For the latter purpose, two different functionalization strategies were investigated for comparing their influence on the sensitivity and stability of the optical sensor response using PrA/rabbit IgG as model system. On the one hand, PrA was directly adsorbed to the metallic surface, and on the other hand, it was covalently attached to the sensor surface using cysteamine/glutardialdehyde. Whereas the shift of the localized surface plasmon resonance is larger for the adsorption of PrA directly to the sensor surface, the stability of the optical response of the sensor is improved in the case of covalently bound PrA. However, both functionalization strategies proved to be sufficient in order to monitor association and dissociation of rabbit IgG to/from PrA and determine accurate equilibrium dissociation constants. Moreover, another type of biomolecular interactions, namely, between ASF/ECL, was also successfully followed in real time using the developed optical sensor. The DL is relatively high in comparison to other reported sensor. However, neither the conditions for recording the reflectance spectra nor the data treatment was optimized for calculating the DLs of the developed sensor. Hence, the DL shown in here are the possible highest values which can be expected for this sensor. For example, an enhancement of the DL may be achieved by using a more sophisticated data treatment—but this is not the objective of this work. In a nutshell, the presented fabrication strategy for plasmonic biosensors is simple and inexpensive and can provide sensitive optical sensors for investigating biomolecular interactions.

## Electronic supplementary material

ESM 1 (PDF 1.00 mb)
