# Supplementary material for: Plasmonic biosensors fabricated by galvanic displacement reactions for monitoring biomolecular interactions in real time
Source: Anal Bioanal Chem. 2020 Jan 31;412(14):3433–45. doi: 10.1007/s00216-020-02414-0 (PMC7214386; doi:10.1007/s00216-020-02414-0)
Supplement: Supplementary file 1 — (PDF 1.00 mb) [file 216_2020_2414_MOESM1_ESM.pdf]

Analytical and Bioanalytical Chemistry

Electronic Supplementary Material

Plasmonic biosensors fabricated by galvanic displacement reactions for monitoring biomolecular interactions in real time

Claudia Pacholski, Sophia Rosencrantz, Ruben R. Rosencrantz,
Ruth Fabiola Balderas-Valadez

1. *Optimization of the nanostructured gold layer*

The reaction of spontaneous galvanic displacement is always occurring when freshly etched porous silicon is immersed in a solution of $HAuCl_4$. By using this reaction continuous gold layers on porous silicon can be formed whose thickness is dependent on the reaction time. These kind of gold layers most often do not show a LSPR peak in their reflectance spectra. However, by carefully choosing the reaction conditions we were able to produce nanostructured gold layers on top of porous silicon which showed a pronounced LSPR in their reflectance spectra. Several parameters were systematically tested in order to obtain nanostructured gold layers with the desired optical properties. These are presented in the following.

The experimental optimization of the fabrication process for obtaining the desired nanostructured gold layers was done using porous silicon prepared from p-type silicon wafers with a resistivity of 0.0006-0.001 Ωcm which were electrochemically etched in a solution of ethanol and HF in a ratio of 1:3 (v:v). The determined optimal parameters were afterwards transferred to silicon wafers with a resistivity of 0.001-0.002 Ωcm which were etched in a solution of ethanol and HF in a ratio of 1:1 (v:v).

1.1. *Content of ethanol in the $HAuCl_4$ solution*

Porous silicon wafers were etched with a current density of 88.5 mA cm^{-2} for 45s and subsequently immersed in solutions of 2mM $HAuCl_4$ in ethanol/water mixtures. The growth of a gold layer was followed by recording specular reflectance spectra and the results are shown in Figure S1.

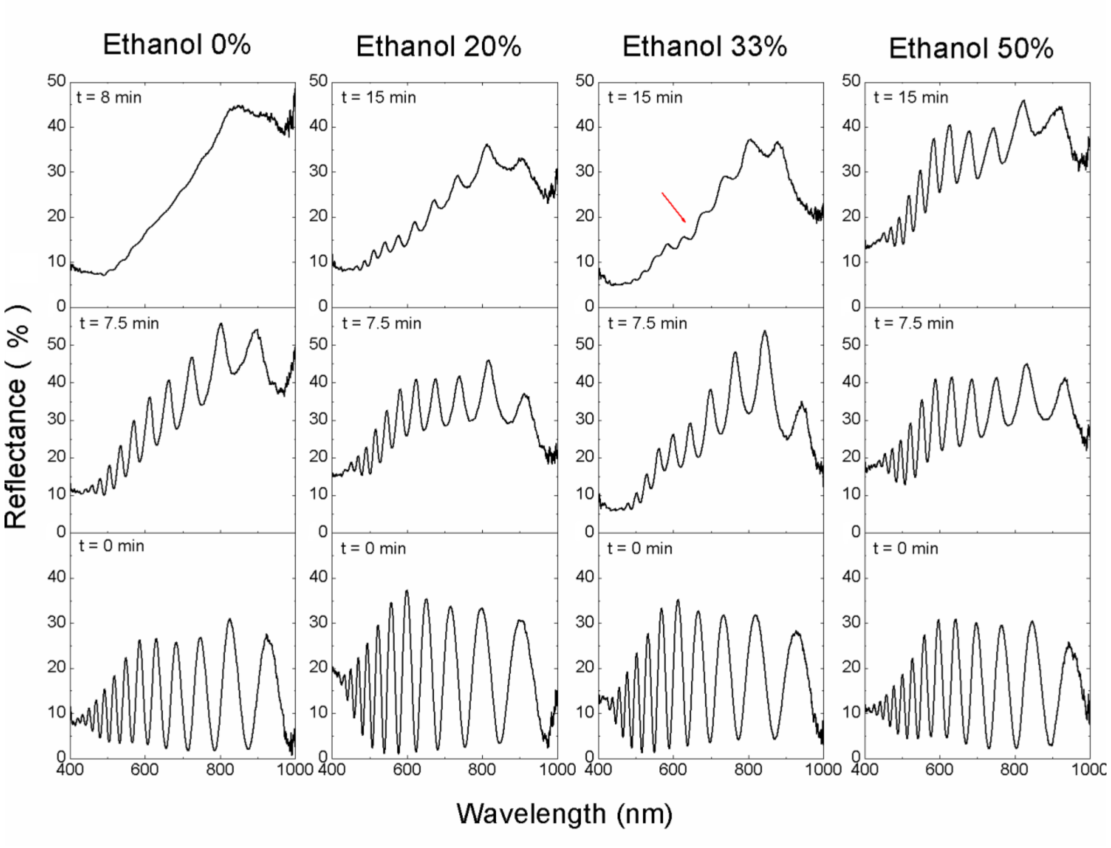

Fig. S1 Optimization of ethanol content in gold solutions. The given time represents the immersion time of the porous silicon in the gold salt solutions. The red arrow indicates the appearance of an optical feature which can be attributed to LSPR

Fresh porous silicon has hydrophobic surface properties affecting the distribution of an aqueous gold salt solution on the porous silicon surface and the infiltration of the solution in the pores. Hence, without ethanol addition to the aqueous gold salt solution the gold layer grows as is shown in Figure S2. This homogenous gold layer is highly reflective and the light cannot reach the porous silicon layer underneath. Consequently, after a reaction time of 8 min (t = 8 min) the fringes of resulting from porous silicon were not visible anymore in the reflectance spectrum. By adding 20% of ethanol to the gold solution the reaction slowed down but a plasmonic feature could not be observed in the reflectance spectra. Increasing the ethanol content to 33% finally led to gold nanostructures with the desired a small plasmonic feature in the reflectance spectra (indicated by the red arrow). Higher ethanol contents of e.g. 50% in the gold solution decreased the reaction speed further and no defined plasmonic feature was observed. It is important to highlight that gold layers prepared using 50 % ethanol in the gold lifted off spontaneously after reaction times of 16-20 min. Hence, the 33% of ethanol was chosen as optimal ethanol content in the aqueous gold solution and other parameters were studied.

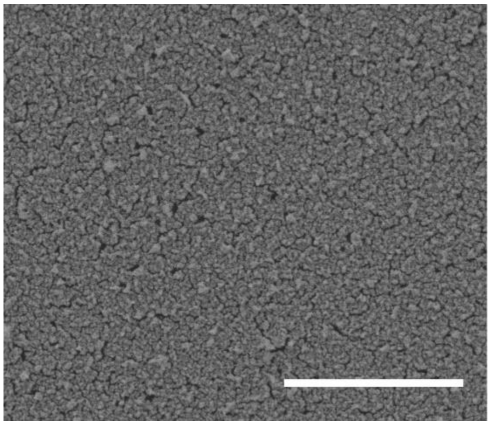

Fig. S2 Micrograph of the gold layer without plasmonic response grown by spontaneous galvanic displacement over fresh etched porous silicon. The impregnation solution did not content any ethanol. (Scale bar = 5µm)

1.2. Porous silicon thickness optimization

The thickness of the porous silicon layer depends on the etching time for the same current density and the same electrolytic solution. Fresh porous silicon samples etched using a current density of 88.5 mA cm^{-1} for different etching time (hence, different thicknesses) were immersed in 2mM $HAuCl_4$ aqueous solution with 33% ethanol for 15 min. The results are shown in Figure S3. The time presented inside the graphs refers to the etching time of the porous silicon layer. A valley in the interference pattern of the porous silicon in the reflectance spectrum was observed in all the samples and traced back to LSPR in the gold layer. However, the resulting nanostructured gold layer from samples with an etching time of 60 s or less were fragile and tended to lift off from the porous silicon surface or to break during the growing process.

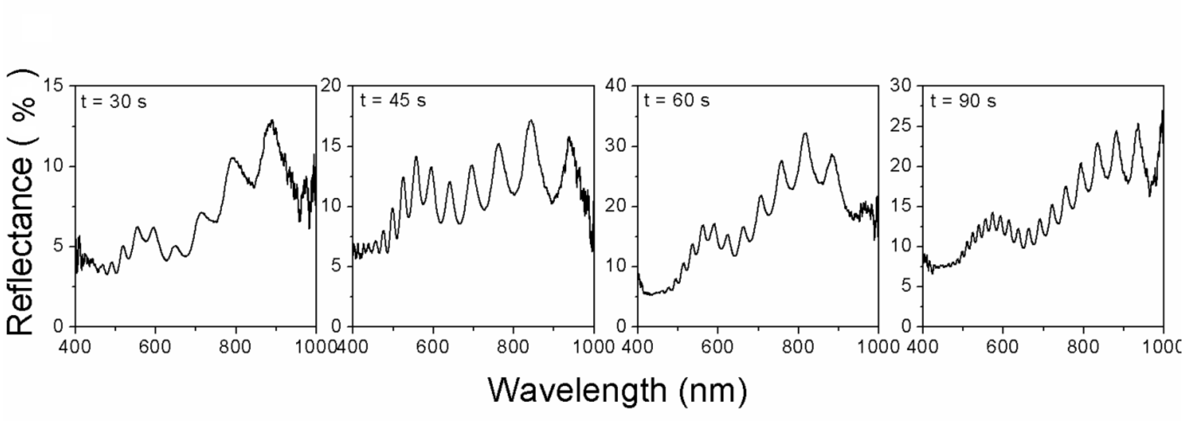

Fig. S3 Optimization of porous silicon thickness. The times given in the graphs represent the etching time of the porous silicon layer

1.3. Pore size optimization

The pore size in a porous silicon layer depends on the current density. For Si wafers having the same resistivity which are etched using the same etching solution, higher current densities lead to the formation of bigger pores. Porous silicon bilayers fabricated using two different current densities in the same etching procedure (one after another) were immersed in the established $HAuCl_4$ solution. In Figure S4 the evolution of the reflectance spectrum of the samples immediately after the immersion in the gold solution (t = 0) and after 15 min (t = 15min) reaction time are displayed. The bilayer presented in Figure S4a was fabricated applying a current density of 265 mA cm^{-1} for 30s and 88.5 mA cm^{-1} for 120 s. The spectrum shown in Figure S4b belong to a bilayer fabricated applying a current density of 496 mA cm^{-1} for 11s and then 168 mA cm^{-1} for 55s. In both cases the pore size on the surface of the bilayers are bigger than in the samples used in the previous sections. However, the valley in the reflectance spectrum caused by LSPR was not obtained. The reduction in the intensity of the fringes is resulting from the gold layer absorbing the light. It is possible to conclude that the range of pores sizes useful for the synthesis of a plasmonic gold layer is smaller and close to the previously used of 88.5 mA cm^{-1}. In this case the pores have a size of ~ less than 20 nm [1].

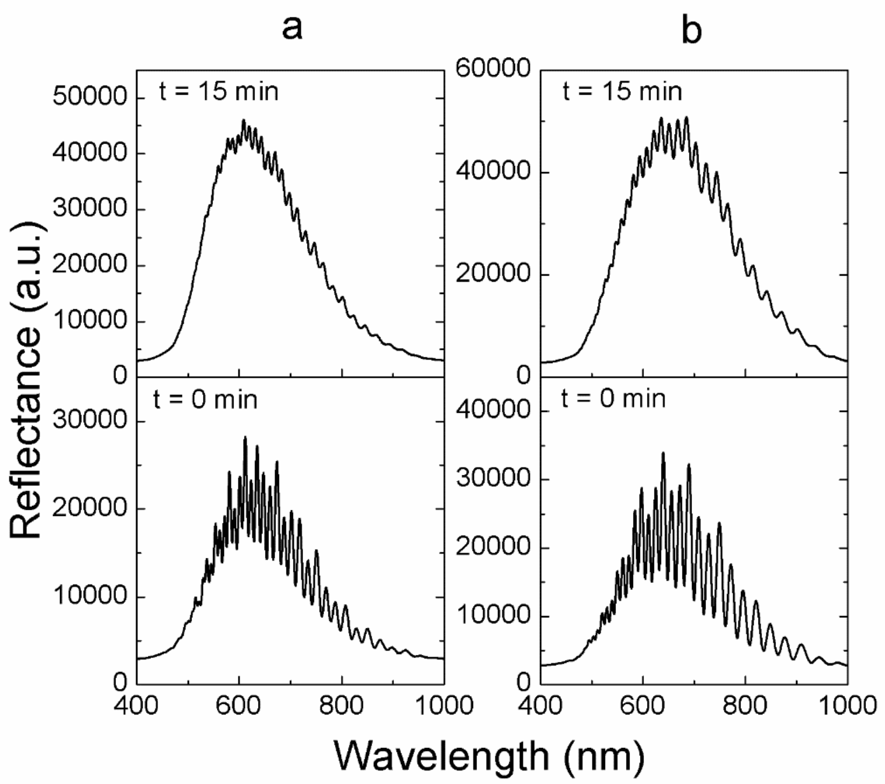

Fig. S4 Effect of the pore size of the porous silicon on the growth nanostructured gold layers. **(a)** bilayer fabricated applying a current density of 265 mA cm^{-1} for 30s and 88.5 mA cm^{-1} for 120 s. **(b)** bilayer fabricated applying a current density of 496 mA cm^{-1} for 11s and then 168 mA cm^{-1} for 55s. (These spectrums were taken without recording a reference spectrum of an aluminum mirror before. Therefore the y-axis (reflectance) is given in arbitrary units.)

1.4. Reaction time

After optimization of the parameters for the fabrication of the porous silicon layer used as template for the growth of nanostructured gold layers the resistivity of the silicon wafer was changed to 0.001-0.002 Ω cm. The pore size, porosity, and the thickness of the porous silicon layers were reproduced in such Si wafers using a calibration curve. However, the usage of different Si wafers in combination with an increased and defined reaction temperature of 31°C reduced the reaction time need to perceive a valley caused by the LSPR in the reflectance spectra to 7 min (Figure S5). Before the change of the silicon wafers and without any temperature control the reaction time required for perceiving LSPR in the reflectance spectrum (valley in the interference patter) was at least 15 min - as was described in sections 1.1 and 1.2 of the supplementary information. Finally, it is important to highlight that the sample should be quickly removed from the gold solution when the valley is visible. Otherwise the gold layer keeps growing and the LSPR response would be lost. Furthermore, the gold layer would lift off from the substrate.

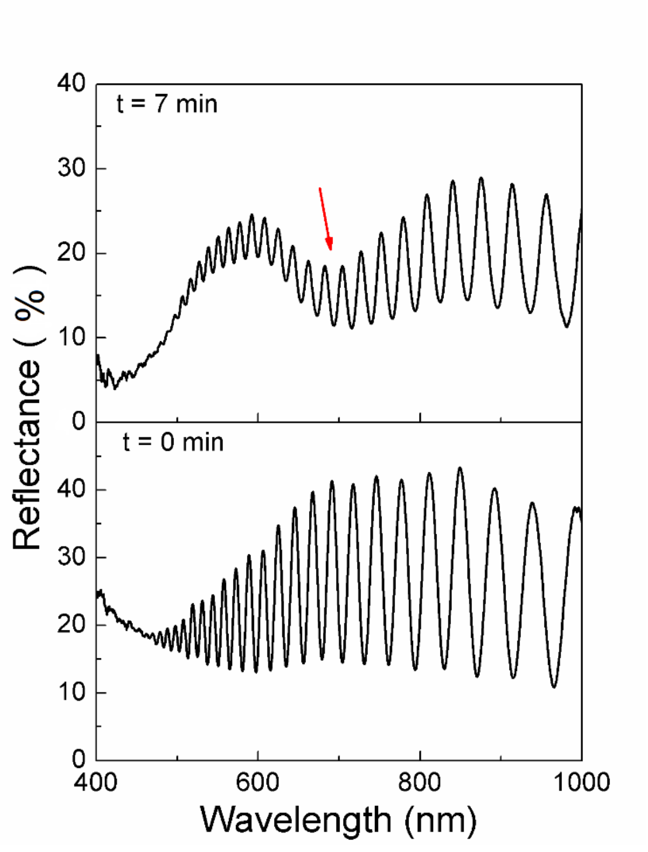

Fig. S5 Reflectance spectra taken from a porous silicon layer (bottom) and a porous silicon layer with a nanostructured gold layer on top (top)

2. Comparation of the nAuL before and after cysteamine functionalization

In order to facilitate their comparison (change in the nanostructures size), we present the two micrographs of the nAuL before and after cysteamine (having the same scale bar) next to each other below (Fig. S6).

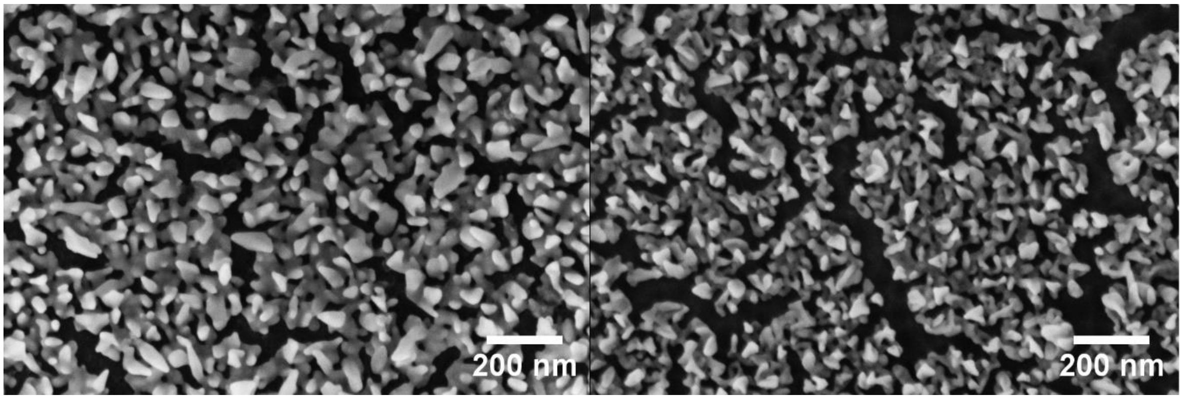

Fig. S6 Scanning electron micrographs of nanostructured gold layers before (left) and after functionalization with cysteamine (right)
